# Involvement of NOS2 Activity on Human Glioma Cell Growth, Clonogenic Potential, and Neurosphere Generation

**DOI:** 10.3390/ijms19092801

**Published:** 2018-09-17

**Authors:** Paola Palumbo, Francesca Lombardi, Giuseppe Siragusa, Soheila Raysi Dehcordi, Sabino Luzzi, AnnaMaria Cimini, Maria Grazia Cifone, Benedetta Cinque

**Affiliations:** 1 Department of Life, Health & Environmental Sciences, University of L’Aquila, Building Delta 6, Coppito, 67100 L’Aquila, Italy; francesca.lombardi@univaq.it (F.L.); giuseppe.siragusa@graduate.univaq.it (G.S.); annamaria.cimini@univaq.it (A.C.); mariagrazia.cifone@univaq.it (M.G.C.); benedetta.cinque@univaq.it (B.C.); 2 Operative Unit of Neurosurgery, San Salvatore Hospital, 67100 L’Aquila, Italy; soheila.raysi@alice.it (S.R.D.); sabino.luzzi@gmail.com (S.L.); 3 Sbarro Institute for Cancer Research and Molecular Medicine and Center for Biotechnology, Temple University, Philadelphia, PA 19122, USA

**Keywords:** NOS2, human glioma cells, 1400W NOS2 inhibitor, tumor cell proliferation and migration, clonogenic potential, neurosphere generation

## Abstract

Aberrant nitric oxide synthase 2 (NOS2) expression has been suggested as an interesting therapeutic target that is being implicated as a component of the molecular profile of several human malignant tumors, including glioblastoma, which is the most aggressive brain tumor with limited therapeutic options and poor prognosis. The aim of the present work was to evaluate the effect of 1400W, a specific NOS2 inhibitor, on human glioma cells in terms of clonogenic potential, proliferation, migration rate, and neurosphere generation ability. NOS2 expression was determined by Western blotting. Nitric oxide (NO) production was measured through nitrite level determination. The trypan blue exclusion test and the plate colony formation assay were performed to evaluate cell proliferation and clonogenic potential. Cell proliferation and migration ability was assessed by the *in vitro* wound-healing assay. Neurosphere generation in a specific stemcell medium was investigated. NOS2 was confirmed to be expressed in both the glioma cell line and a human glioma primary culture, and overexpressed in relative derived neurospheres. Experiments that aimed to evaluate the influence of 1400W on U-87 MG, T98G (glioblastoma cell lines) and primary glioma cells sustained the crucial role played by NOS2 in proliferation, colony formation, migration, and neurosphere generation, thus supporting the emerging relevance of a NOS2/NO system as a prognostic factor for glioma malignancy and recurrence.

## 1. Introduction

Glioblastoma (GBM), the most malignant adult brain tumor, is characterized by a high proliferative and invasive rate with very limited therapeutic options and poor prognosis [1,2,3]. Despite multiple studies that have aimed to define the potential determinants of the aggressiveness of this tumor, the mechanisms of GBM genesis remain undefined, and the molecular factors underlying its aggressiveness are still unclear [4,5]. The current pathophysiological hypothesis involves so-called glioma stem cells (GSCs) being responsible for the formation, expansion, recurrence, and the high therapy resistance of GBM [6,7,8,9,10]. The ability to promote glioma relapse, angiogenesis, invasiveness, and therapeutic resistance renders GSCs a potential target for anti-GBM therapy [11,12,13]. Several studies have suggested that gliomas, similar to most established malignant tumors, are characterized by a moderately inflammatory environment. The inflammatory process seems to be involved in all of the steps of tumorigenesis, promoting the genomic instability, proliferation, and survival of malignant cells, as well as angiogenesis, resistance to therapy, local or systemic immunosuppression, and also raising the metastatic process [14,15,16,17,18,19]. Aberrant nitric oxide synthase 2 (NOS2) expression and its enzymatic product nitric oxide (NO), which play a crucial role in the pathophysiology of several inflammatory disorders, have been implicated in the development, growth and progression of several human malignant tumors, including glioma [20,21,22]. NOS2 has been reported highly expressed in grade III astrocytomas and glioblastomas, with a positive correlation between its expression and tumor grade [20]. As recently reviewed [23], an overexpressed NOS2/NO system in the tumor cell induces invasion, angiogenesis, immunosuppression, differentiation, and therapeutic resistance in gliomas. GSCs have been shown to express high NOS2 levels, which were correlated with a poorer glioma patient survival [24]. Furthermore, the silencing of NOS2 expression by RNA interference decreased *in vitro* brain glioma-initiating cells (GICs), highlighting the main role of NOS2 in GSC biology and maintenance [24]. NOS2 knockdown by RNA interference strategy or by specific inhibitors negatively affected the proliferation and invasiveness of GBM cells [20,25], and was able to reduce the progression of subcutaneous and intracranial human glioma xenografts in mice [24]. The increase or the significant inhibition of tumor cell migration were respectively recorded after treating a co-culture of U87-MG and C6 glioma cell lines with the NO-donor sodium nitroprusside (SNP), or the NOS inhibitor NAME (Nω-nitro-l-arginine methyl ester) [26]. The key roles of NOS2 in tumor development and vessel maturation in the C6 rat glioma cell line were also published [27]. In a recent study, our group reported that NOS2 expression was highly and significantly upregulated in glioma cells that were kept in the specific medium for neurosphere generation [28]. Moreover, a high and significant correlation was observed among the expression of NOS2 and SOX-2 (Sex determining region Y-box 2), which is a stemness marker that is aberrantly upregulated in both human glioma cell lines and primary cultures.

NOS2 pharmacological inhibition might therefore have potential therapeutic value in the treatment of GBM. A major class of NOS2 inhibitors are amidine derivatives, such as L-NIL, the cyclic amidine ONO-1714, and the aromatic acetamidine 1400W [29]. This latter is considered to be one of the most potent and selective NOS2 inhibitors reported to date [29,30,31], although it has never been approved into clinical use. Pharmacokinetic studies showed that 1400W is an irreversible or an extremely slowly reversible inhibitor of NOS2, although it has been reported to be active for a few hours after administration [32,33]. In the continuous effort to develop even more selective and effective NOS2 inhibitors, different acetamidines structurally related to the 1400W leading scaffold have been published [31,34,35,36,37], thus confirming the growing interest in the pharmacologic potential of NOS2 activity inhibition in different diseases, including GBM.

In the present study, the NOS2 expression and activity in the U-87 MG cell line and human GBM primary cells have been analyzed. To verify the potential functional role of NOS2 activity in glioma biology, the effects of the addition of 1400W were evaluated in the proliferation and migration rate, clonogenic potential, and capacity of generating neurospheres of both GBM cell line and primary cells. To investigate the involvement of an exogenous NO on these cell systems, in some experiments, the NO chemical donor S-nitroso-N-acetylpenicillamine (SNAP) was also used.

## 2. Results

### 2.1. NOS2 Expression and Activity in Adherent U-87 MG Cell Line

**Figure 1:**
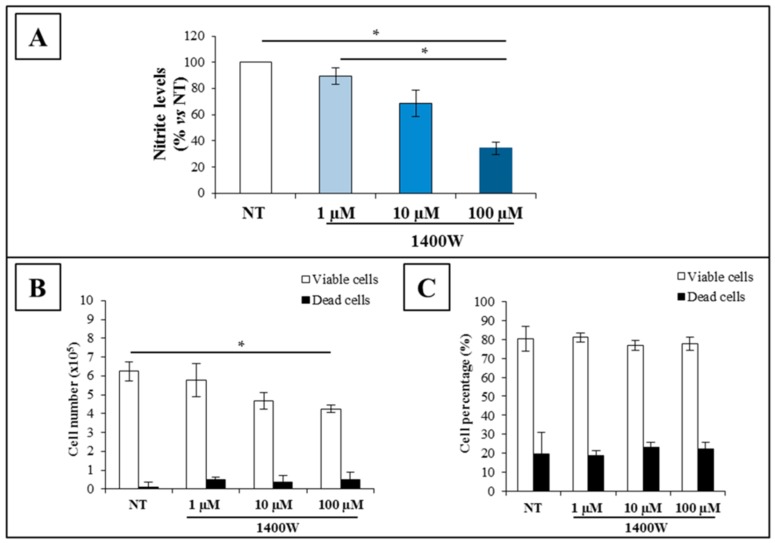
Effect of different concentrations of 1400W, a nitric oxide synthase 2 (NOS2) inhibitor, on U-87 MG human glioblastoma (GBM) cell line. (**A**) Nitrite levels in media from adherent U-87 MG cells cultured for 24 h without (not treated, NT) or with NOS2 inhibitor, 1400W (1 µM,10 µM, and 100 µM). The results presented as percentage vs. NT are expressed as the mean ± SEM from two independent experiments in triplicate. (**B**) U-87 MG cells were treated with 1400W at 1 μM, 10 μM,100 μM, or PBS (NT) for 24 h, and the number of viable and dead cells was assessed by Trypan blue exclusion test and (**C**) by propidium iodide (PI) staining. Results are presented as the mean ± SEM of two independent experiments in triplicate. For comparative analysis of groups of data, one-way ANOVA followed by Bonferroni post hoc test was used (* *p* < 0.05).

To examine the effect of the well-known inhibitor of NOS2, 1400W, on the human U-87 MG cell line, the more effective concentration to inhibit NOS2 activity has been firstly evaluated by a dose-response curve. The cells were treated with different concentrations (1 μM, 10 μM, and 100 μM) of 1400W for 24 h, and the NOS2 enzymatic activity, which was evaluated as nitrite levels, was assayed in the culture medium. In Figure 1A, the results expressed as percentage versus NT are presented. The addition of a NOS2 inhibitor at 100 μM significantly reduced the basal nitrite levels evaluated after 24 h (*p* < 0.05 vs. NT and 10 μM) in U-87 MG culture. Considering the high specificity of the NOS2 inhibitor 1400W, the amount of nitrites that reduced by 1400W should be attributable to the NOS2 activity. The cell number appeared to be significantly reduced by the incubation of 100 µM 1400W for 24 h, as analyzed by the Trypan blue dye exclusion test (*p* < 0.05). It is of note that the dead cell number was not influenced by any different concentration of 1400W (Figure 1B). The analysis of the percentage of U-87 MG dead cells, as assessed through propidium iodide (PI) staining by cytofluorometry, confirmed that the 1400W inhibitor did not cause a cytotoxic effect at all used concentrations (Figure 1C). A considerable decrease in the cell number and nitrite levels of 1400W was obtained with 100 µM; hence, the following experiments were performed with the single 1400W concentration (100 µM), as previously reported [24]. For these experiments, RAW 264.7 cells that were either unstimulated or stimulated with LPS and IFN-γ were used as the negative and positive control, respectively. Also, for this cell line, no cytotoxic effect was detected when treated with 1400W for up to 100 μM(data not shown).

**Figure 2:**
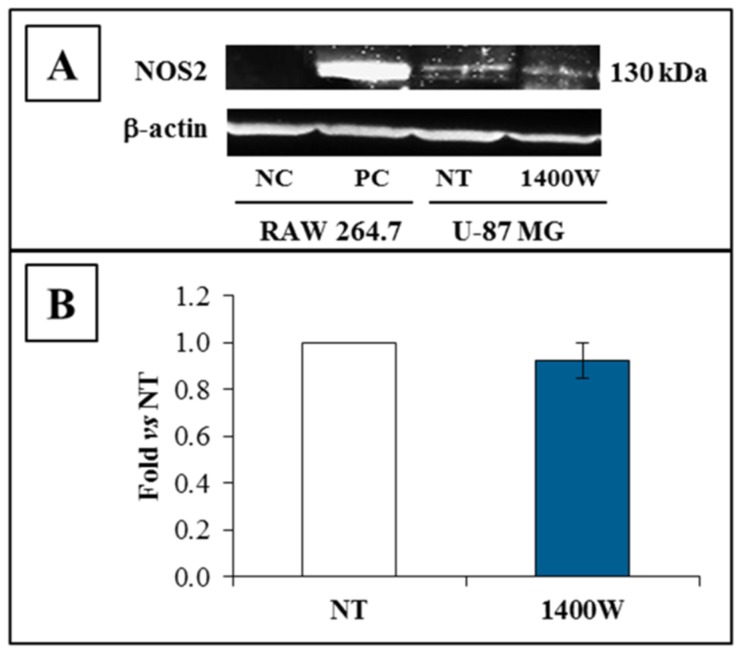
NOS2 protein expression of the adherent U-87 MG glioblastoma cell line. (**A**) Representative Western blot of NOS2 and β-actin. U-87 MG cells were cultured and treated or not with 1400W (100 µM) as described for 24 h. NC (negative control): Untreated RAW 264.7; PC (positive control): RAW 264.7 treated with LPS (Lipopolysaccharide) (1 µg/mL) and IFN-γ (Interferon-γ) (100 ng/mL) for 24 h. (**B**) Quantification analysis of blots by densitometry is expressed as fold increase vs. NT. The results from three independent experiments in duplicate are presented and expressed as mean ± SEM.For comparison between two means, Student’s unpaired *t*-test was used.

To further confirm our previous results obtained with RT-PCR [28], NOS2 protein levels were firstly evaluated in adherent U-87 MG cells after 24 h of culture. As expected, U-87 MG cells basally expressed NOS2 protein, and the exposure to the 1400W inhibitor did not affect the NOS2 protein expression, as evident in the representative Western blot shown in Figure 2A. The NOS2 protein levels expressed by fold versus those not treated (NT) are presented in Figure 2B.

### 2.2. NOS2 Inhibition Strongly Affects the Proliferation Rate of an Adherent U-87 MG Cell Line

**Figure 3:**
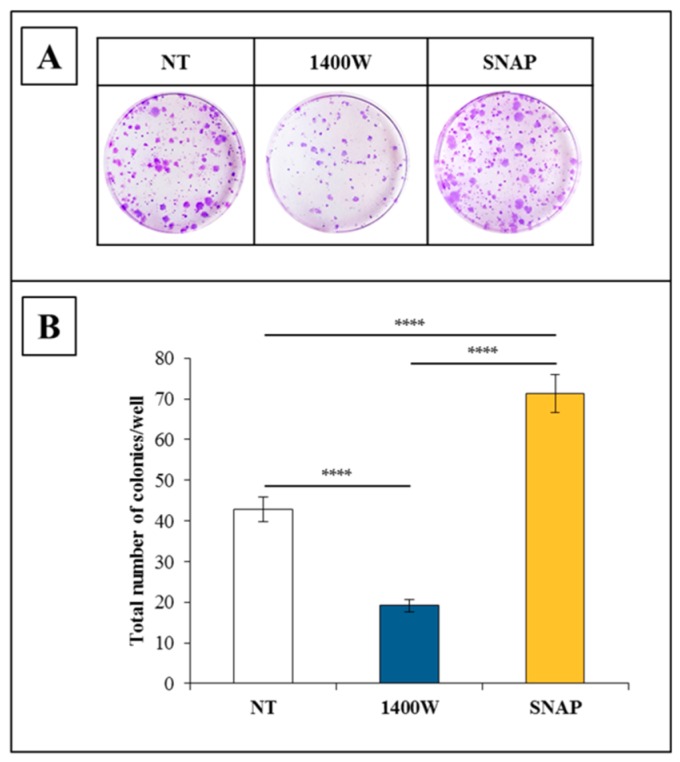
Nitric oxide is involved in the clonogenic potential of U-87 MG cells. (**A**) Representative microscopic images of clonogenic assay of U-87 MG cells after daily treatment for 10 days with NOS2 activity inhibitor 1400W (100 µM) or nitric oxide(NO)-donor S-nitroso-N-acetylpenicillamine (SNAP) (100 µM). NT = not treated. (**B**) Quantitative results of clonogenic assay expressed as total number of surviving colonies/well. Viable colonies with diameter >0.3 mm were counted with an ocular micrometer (mean ± SEM of three independent experiments in triplicate). For comparative analysis of groups of data, one-way ANOVA followed by Bonferroni post hoc test was used (**** *p* < 0.0001).

The clonogenic potential of U-87 MG treated daily with 1400W for 10 days was significantly lower (*p* < 0.0001) than that in the control group, which indicated the crucial role of NOS2 activity on the capacity of the glioma cell line to produce progeny. On the other hand, NO-donor SNAP (100 μM) led to an improved clonogenic potential when compared to control cells (*p* < 0.0001 vs. not treated cells and 1400W-treated cells) (Figure 3B). In Figure 3A, representative microscopy images of clonogenic assay are presented, while in Figure 3B, the results expressed as the number of colonies/well (mean ± SEM of three independent experiments performed in triplicate) are shown. 

**Figure 4:**
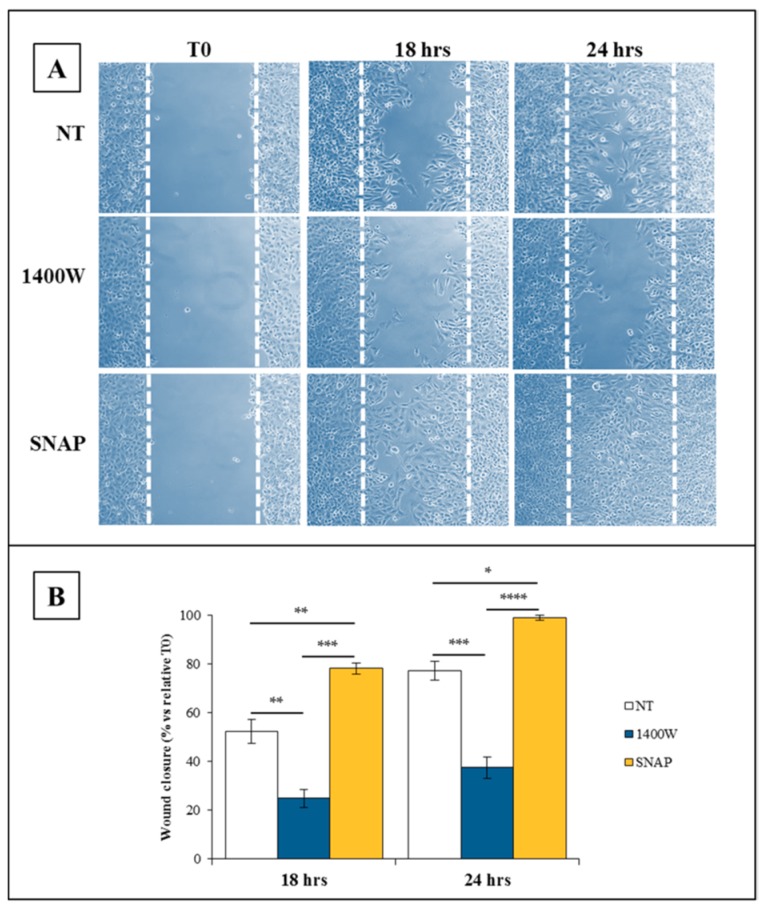
Involvement of NO on the scratch-wound healing ability of U-87 MG cells. (**A**) Representative microscopy images of the scratch-wound healing assay captured at 0 h, 18 h, and 24 h. Scratched U-87 MG monolayers were incubated without (not treated, NT) or with NOS2 inhibitor 1400W (100 µM) or NO-donor SNAP (100 µM) for the indicated times after injury (10× magnification). (**B**) The extent of the wound closure rate was calculated as described and expressed as % closure versus relative T0 at 18 h and 24 h. Data are expressed as the mean ± SEM of two independent experiments in duplicate. For a comparative analysis of groups of data, repeated measures two-way ANOVA followed by a Bonferroni post hoc test was used (* *p* < 0.05, ** *p* < 0.01, *** *p* < 0.001, **** *p* < 0.0001).

As invasiveness is one of the pathophysiological features of human GBM, to support the functional role of the NOS2/NO system in our cell system, the migration and proliferation abilities of U-87 MG cells cultured at different time intervals with or without 1400W (100 μM) were also checked through an *in vitro* scratch wound assay. At 36 h, a scratched monolayer of not treated U-87 MG cells was mainly closed (not shown). The effect of NO-donor SNAP (100 μM) was also evaluated to verify the ability of NO to positively influence both migration and proliferation rate in our cell model. The results showed that the migration and proliferation abilities of U-87 MG cells were strongly and significantly inhibited by an NOS2 inhibitor, as evident in the representative microscopy images of the scratch-wound healing assay captured at 0 (T0), 18 h, or 24 h, as shown in Figure 4A. The results of a quantitative analysis of the wound-closure rate from two independent experiments in duplicate are reported in Figure 4B. Data are expressed as the % closure rate of a scratched monolayer at 18 h and 24 h versus relative T0 (mean ± SEM). For the comparative evaluation of groups of data, a repeated measure two-way ANOVA followed by Bonferroni post hoc test was performed. The addition of 1400W significantly and negatively affected the closure rate of a scratched U-87 MG monolayer both at 18 h (*p* < 0.001) and at 24 h (*p* < 0.0001). This finding could be due to the effect on cell proliferation even if a slowed cell migration could not be excluded. Accordingly with the results above described, treatment with NO-donor SNAP was able to strongly and positively influence the basal wound closure rate either at 18 hours or 24 hours (*p* < 0.01 and *p* < 0.001 vs. not treated monolayers, respectively).

### 2.3. NOS2 Expression and Activity in U-87 MG-Derived Neurospheres

**Figure 5:**
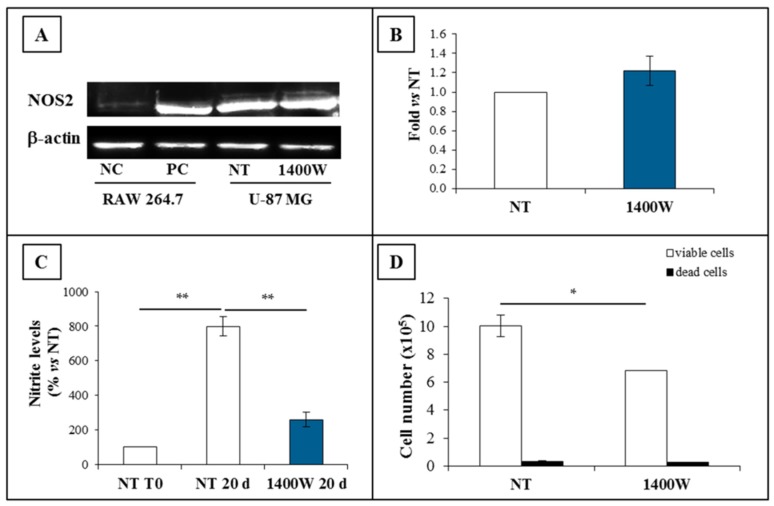
NOS2 protein expression and activity in U-87 MG-generated neurospheres. (**A**) Representative Western blot of NOS2 and β-actin. U-87 MG neurospheres were incubated in the absence (not treated, NT) or presence of 1400W (100 µM) daily added for 20 days. NC (negative control): untreated RAW 264.7; PC (positive control): RAW 264.7 treated for 24 h with LPS (1 µg/mL) and IFN-γ (100 ng/mL). (**B**) Quantification analysis of blots by densitometry expressed as fold vs. NT. The results from three independent experiments in duplicate are presented and expressed as mean ± SEM. (**C**) Nitrite levels in U-87 MG neurospheres’ media in the absence or presence of NOS2 inhibitor, with 1400W (100 µM) daily added for 20 days. The nitrite levels at T0 are also shown. Data are expressed as percentage of nitrite levels vs. NT (mean ± SEM of two independent experiments in duplicate). For comparative analysis of groups of data, a two-way analysis of variance (ANOVA) with post hoc Bonferroni test was used (** *p* < 0.01). (**D**) Effect of NOS2 inhibitor 1400W (100 µM) on U-87 MG-generated neurosphere viability after 20-day culture. Data are expressed as the mean ± SEM of two independent experiments performed in duplicate. For comparison between two means, Student’s unpaired *t*-test was used (* *p* < 0.05).

NOS2 expression and activity were also investigated in neurospheres. The results of Western blot analysis showed that the NOS2 protein was overexpressed in U-87 MG-generated neurospheres at 20 days of culture, thus confirming our previous results from the RT-PCR assay of NOS2 [28]. The 1400W addition to the cell culture did not influence the expression levels of NOS2 when compared to neurospheres that were not treated (Figure 5A).The results that were designed to evaluate the NOS2 protein fold change versus NT are shown in Figure 5B. To investigate the involvement of NOS2 activity in the neurosphere generation, U-87 MG cells were cultured in GSC-M condition in the presence or absence of 1400W (100 μM). As shown in Figure 5C, a strong increase of NOS activity was evaluated as nitrite levels occurred at 20 days when compared to basal levels at T0 (*p* < 0.01). On the other hand, nitrite levels were drastically reduced by the presence of 1400W (*p* < 0.01 as compared to NT at 20 days), and were not significantly different versus NT at T0. As observed in U-87 MG cells cultured in standard medium (St-M), NOS2 inhibitor was also able to inhibit the cell growth of neurosphere-forming cells in GSC-M, (*p* < 0.05 versus not treated neurospheres) without affecting cell viability, which registered >95% (Figure 5D).

### 2.4. NOS2 Inhibition Strongly Affects the Ability of U-87 MG Cells to Generate Neurosphere

**Figure 6:**
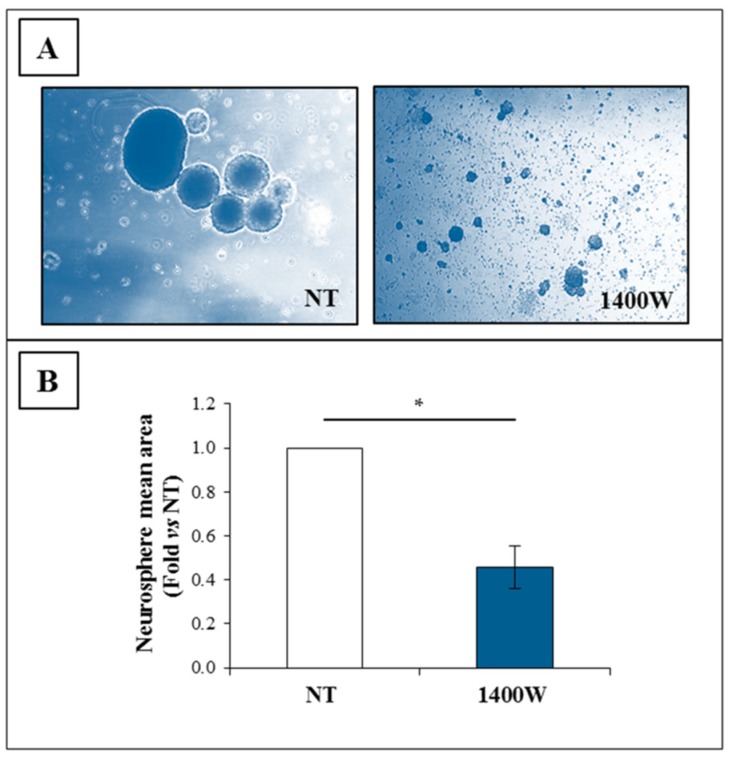
Nitric oxide synthase (NOS) activity inhibition strongly affects U-87 MG neurosphere generation. (**A**) Representative phase contrast images (4× magnification) of human U-87 MG cell line maintained in glioma stem cells condition (GSC-M) to allow neurospheres’ generation in the absence (not treated, NT) or presence of NOS2 activity inhibitor 1400W (100 µM), added daily for 20 days. Baseline condition (NT) at five-day culture is shown. (**B**) Quantification analysis of neurosphere mean area expressed as fold vs. NT. Data represent mean ± SEM of two independent experiments in duplicate. For comparison between two means, Student’s unpaired *t*-test was used (* *p* < 0.05).

The ability of U-87 MG cells to generate neurospheres, which is a surrogate marker of GSC self-renewal, was also significantly affected by the presence of 1400W at 20 days of incubation, thus supporting the functional role of the NOS2/NO system in the sphere-forming ability of tumor stem cells. Representative images captured with the phase contrast microscope are shown in Figure 6A. The data of neurosphere mean area expressed as fold versus NT are shown in Figure 5B as mean ± SEM (*p* < 0.05).

### 2.5. Effect of 1400W Inhibition on T98G Cell Line and Respective Neurospheres

**Figure 7:**
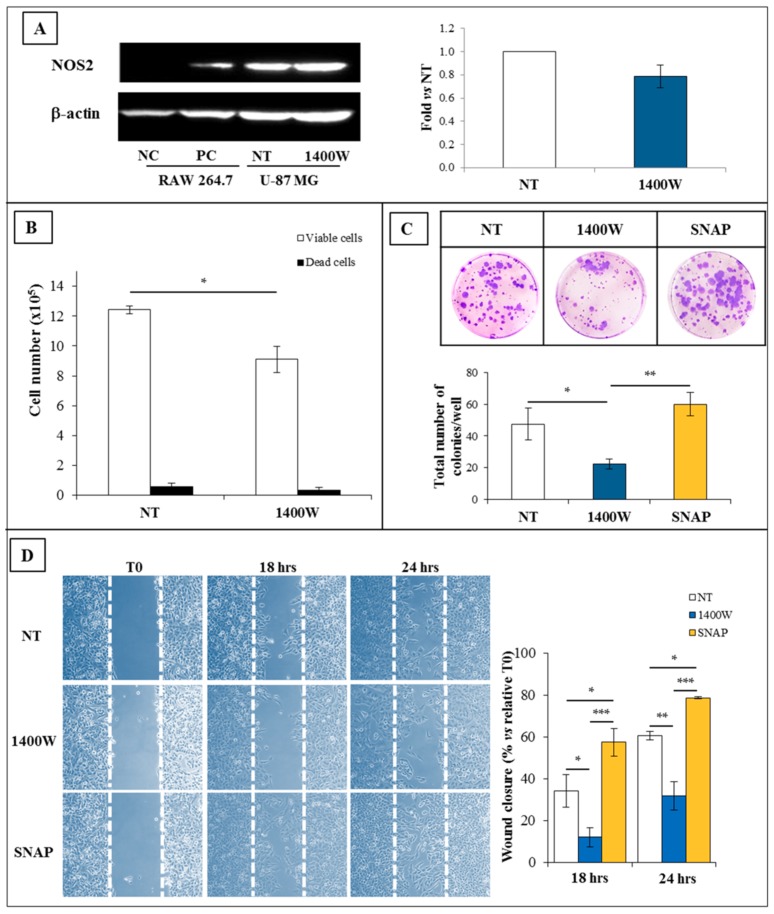
Expression and functional role of NOS2 in adherent human T98G cell line. (**A**) Representative Western blot of NOS2 and β-actin in T98G cells cultured in standard medium, (St-M) for 24 h. NC (negative control): Untreated RAW 264.7; PC (positive control): RAW 264.7 treated with LPS (1 µg/mL) and IFN-γ (100 ng/mL) for 24 h. Results of quantification analysis of blots by densitometry expressed as fold vs. NT are reported in the histogram. The results from one experiment in duplicate are presented as mean ± SD. (**B**) Effect of NOS2 inhibitor 1400W (100 µM) on T98G cell number (viable and dead cells) after 24 h of culture. The results from one experiment in duplicate are showed as mean ± SD. For comparison between two means, Student’s unpaired *t*-test was used (* *p* < 0.05). (**C**) Representative microscopic images of clonogenic assay of T98G cells not treated (NT) or treated daily with NOS2 activity inhibitor 1400W (100 µM) for 10 days. The quantitative results of clonogenic assay are reported in the histogram and expressed as the total number of surviving colonies/well. Data represent the mean ± SD of one experiment in duplicate. For comparative analysis of groups of data, repeated two-way ANOVA measures followed by a Bonferroni post hoc test were used (* *p* < 0.05, ** *p* < 0.01). (**D**) Representative microscopy images of the scratch-wound healing assay captured at 0, 18 h, and 24 h. Scratched T98G monolayers were incubated without (NT) or with NOS2 inhibitor 1400W (100 µM) or NO-donor SNAP (100 µM) for the indicated times after injury (10× magnification). The wound-closure rate was calculated as described and expressed as % closure vs. relative to T0 at 18 h and 24 h. Data are expressed as the mean ± SD of one experiment in duplicate. For the comparative analysis of groups of data, repeated measures two-way ANOVA followed by a Bonferroni post hoc test were used (* *p* < 0.05, ** *p* < 0.01, *** *p* < 0.001).

**Figure 8:**
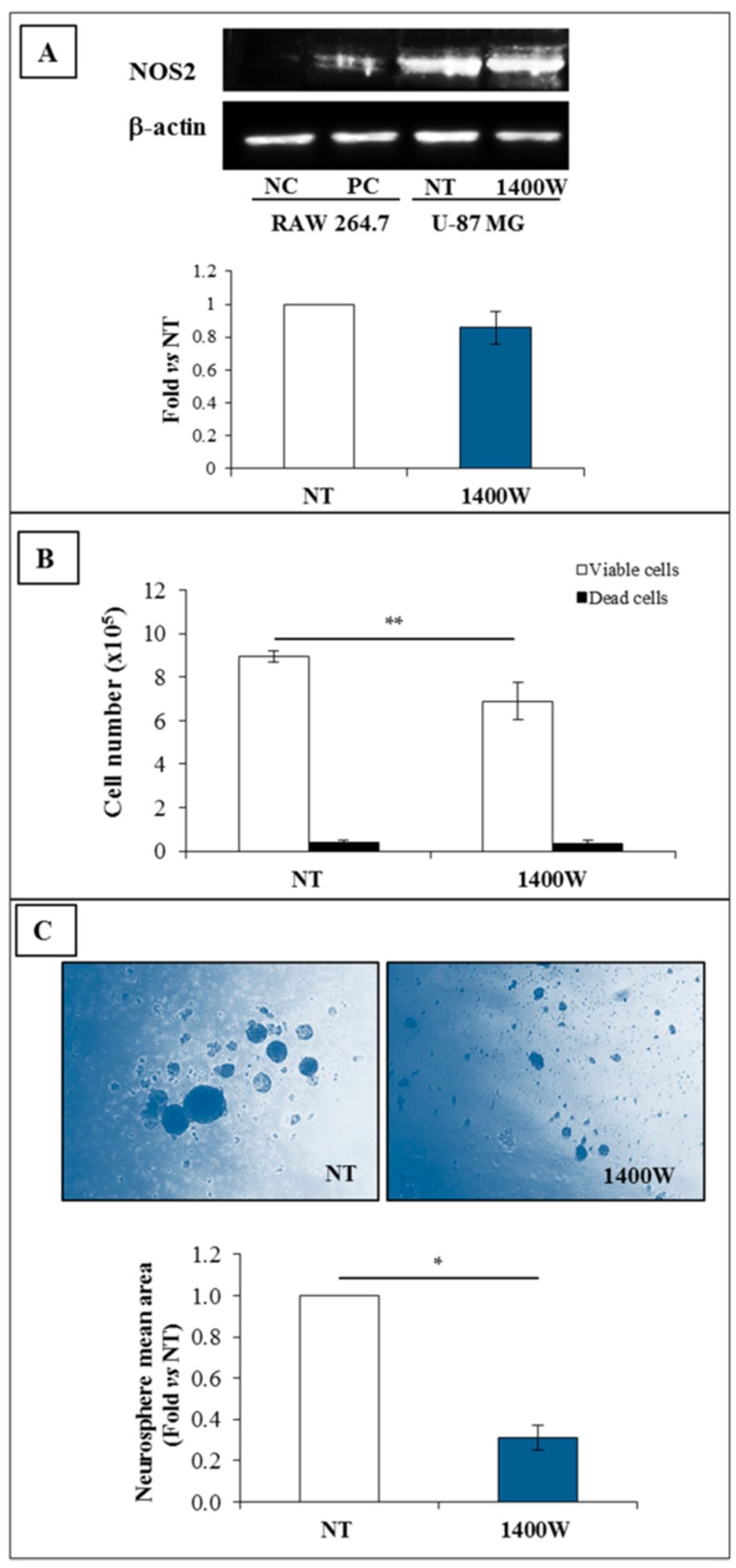
NOS2 expression in T98G-derived neurospheres. (**A**) Representative Western blot of NOS2 and β-actin of T98G-generated neurospheres not treated (NT) or treated with 1400W (100 µM) which was daily added to the culture for 20 days. NC (negative control): Untreated RAW 264.7; PC (positive control): RAW 264.7 treated with LPS (1 µg/mL) and IFN-γ (100 ng/mL) for 24 h. Results of quantification analysis of blots by densitometry, expressed as fold vs. NT, are reported in the histogram and relative to one experiment in duplicate (mean ± SD). For comparison between NT and 1400W conditions, Student’s unpaired *t*-test was used (not significant). (**B**) Effect of NOS2 inhibitor 1400W (100 µM) on the cell number of T98G neurospheres after 24 h of culture. The results from one experiment in duplicate are shown as mean ± SD. For comparison between two means, a Student’s unpaired t-test was used (** *p* < 0.01). (**C**) Representative phase contrast images (4× magnification) of T98G neurospheres in the absence (NT) or presence of 1400W (100 µM) added daily added for 20 days. The quantification analysis of neurosphere mean area reported as fold vs. NT is shown in the histogram and expressed as the mean ± SD of one experiment in duplicate. For comparison between two means, Student’s unpaired *t*-test was used (* *p* < 0.05).

To verify the sensitivity to 1400W, another GBM cell line, T98G, was also exposed to NOS2 inhibitor 1400W at 100 μM for 24 h. Obtained results were consistent with data from U-87 MG experiments. Figure 7 and Figure 8 show the effect of 1400W on adherent T98G and derived neurospheres, respectively. Similar to U-87 MG, treatment with 1400W did not induce a decrease of NOS2 protein levels both in adherent cells and neurospheres (Figure 7A and Figure 8A, respectively), while it was able to negatively affect the viable cell number not showing cytotoxic effect (Figure 7B and Figure 8B, respectively). Moreover, the clonogenic potential of adherent T98G, as well as the wound closure rate, were also significantly affected by 1400W exposure (Figure 7C,D, respectively). The mean area of T98G-derived neurospheres daily treated with 1400W and detected after 20 days was significantly reduced (Figure 8C).

### 2.6. Involvement of NOS2 on the Clonogenic Potential and Ability to Generate Neurospheres of Glioma Primary Cells

**Figure 9:**
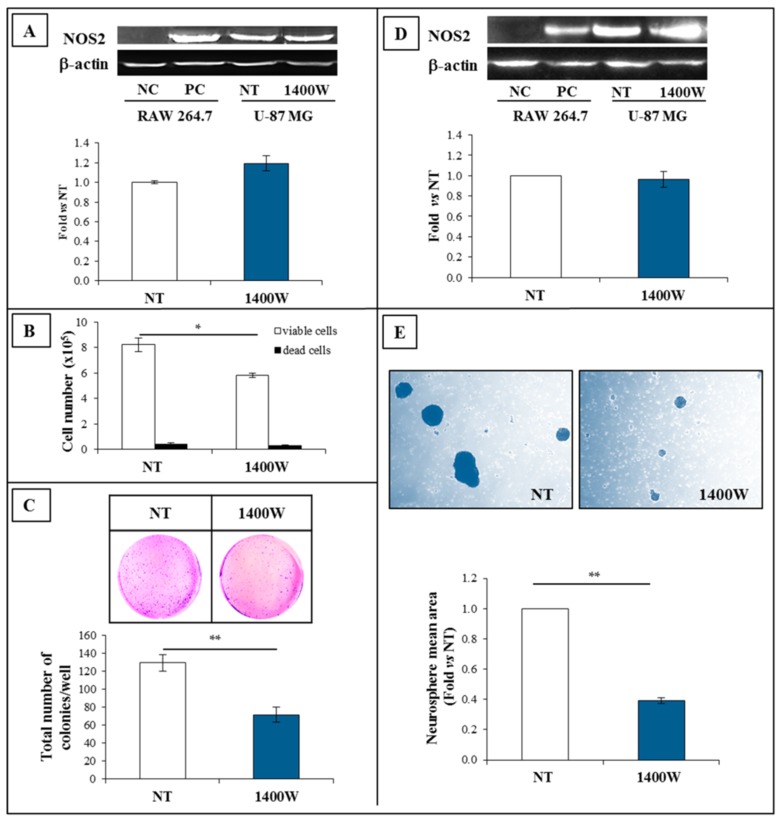
NOS2 expression in human glioblastoma primary cultures. (**A**) Representative Western blot of NOS2 and β-actin in GBM adherent glioma primary cells cultured in standard condition for 24 h. NC (negative control): Untreated RAW 264.7; PC (positive control): RAW 264.7 treated with LPS (1 µg/mL) and IFN-γ (100 ng/mL) for 24 h. Results of quantification analysis of blots by densitometry expressed as fold vs. NT are reported in the histogram. The results from two independent experiments in duplicate are presented as mean ± SEM. (**B**) Effect of NOS2 inhibitor, 1400W (100 µM) on GBM adherent glioma primary culture cell number (viable and dead cells) after 24 h of culture. The results from two independent experiments in duplicate are presented as mean ± SEM. For comparison between two means, Student’s unpaired *t*-test was used (* *p* < 0.05). (**C**) Representative microscopic images of the clonogenic assay of human adherent GBM primary culture not treated (NT) or treated daily with NOS2 activity inhibitor 1400W (100 µM) for 20 days. Quantitative results of clonogenic assay are reported in the histogram and expressed as the total number of surviving colonies/well. Data represent the mean ± SEM of two independent experiments in duplicate. For comparison between two means, Student’s unpaired t-test was used (** *p* < 0.01). (**D**) Representative Western blot of NOS2 and β-actin in primary culture-generated neurospheres not treated (NT) or treated with 1400W (100 µM), which was daily added to the culture for 20 days. NC (negative control): Untreated RAW 264.7; PC (positive control): RAW 264.7 treated with LPS (1 µg/mL) and IFN-γ (100 ng/mL) for 24 h. Results of quantification analysis of blots by densitometry, expressed as fold vs. NT are reported in the histogram. The results from three independent experiments are presented as mean ± SEM. For comparison between NT and 1400W conditions, Student’s unpaired *t*-test was used. (**E**) Representative phase contrast images (4× magnification) of human GBM primary culture maintained in GSC-M condition (GSCs) to allow neurosphere generation in the absence (not treated, NT) or presence of 1400W (100 µM), added daily for 20 days. Quantification analysis of neurosphere mean area reported as fold vs. NT is shown in the histogram and expressed as the mean ± SEM of two independent experiments in duplicate. For comparison between two means, Student’s unpaired *t*-test was used (** *p* < 0.01).

The involvement of NOS2 activity was also investigated in the human glioma primary cells. As observed in the U-87 MG and T98G cell lines, glioma primary cells cultured in St-M basically expressed NOS2 (Figure 9A). The addition of 1400W strongly and negatively affected both the cell growth and colony formation (*p* < 0.05 and *p* < 0.01versus not treated control cells, respectively) (Figure 9B,C). However, 1400W did not influence cell viability (>95% viable cells). NOS2 protein resulted in overexpressed neurospheres generated by the primary glioma cells after 20 days of culture in GSC-M as compared to relative adherent cells cultured in St-M. Also in this case, no effect on NOS2 protein levels was evidenced after the daily addition of 1400W for 20 days (Figure 9D). On the other hand, neurosphere generation by primary glioma cells was strongly and significantly reduced by 1400W treatment, either in terms of number or size. In Figure 9E, representative images are shown captured with phase contrast microscope together data of neurosphere mean area expressed as mean ± SEM (*p* < 0.01 as compared to NT at 20 days). 

## 3. Discussion

Despite advances in therapeutic approaches, the poor prognosis of patients with GBM has not improved, with a mean survival of ~15 months. Thus, new therapeutic stratagems that can successfully suppress GBM are urgently required. Actually, GSCs are mainly considered to be responsible for high resistance to therapies and tumor recurrence. NOS2 was shown to play an important role in GSC survival, proliferation, stemness expression, and therapy resistance. Fractionated radiotherapy has been reported to increase SOX2 and Notch expression through NOS2/NO system upregulation, leading to glioma resistance to radiotherapy [25]. NO signaling has been involved in the ID4 (inhibitor of differentiation-4)-induced enhancing effect on SOX2 expression and the resistance of GSC to chemotherapy [38,39]. NOS2 expression was shown to promote the induction of stem cell properties in GBM cells [40]. The positive regulatory circuit that is associated with platelet-derived growth factor (PDGF)–NO–ID4 signaling has been suggested to play a pivotal role in regulating the self-renewal and glioma-initiating cells (GICs) [40]. According to a recent article, the upmodulation of NOS2, among other inflammatory enzymes (i.e., COX2), is associated with cell survival mechanisms during an oxidative stress condition by which GBM cells adapted themselves to aberrant metabolic activities [41]. A crucial role of the NOS2/NO system in immunosuppression has also been evidenced [42,43]. Recently, in a model system of GBM—non-ionizing photodynamic therapy—the reported findings indicated that suppressing NOS2 expression markedly increased the efficacy of anti-tumor therapy [44]. Recently, the ability of temozolomide (TMZ), alone or in combination with thymoquinone (TQ), to reduce NO production by U-87 MG cells has been suggested as a potential mechanism underlying its anti-glioma activity [45,46]. The pharmacological inhibition of NOS by l-NMMA (N^G^-Monomethyl-l-arginine) has been reported to enhance chemotherapy response in triple negative breast cancer (TNBC) models, significantly reducing tumor volume growth and increasing the survival rate [47]. Our group has recently shown that the basal levels of NOS2/NO system expression significantly increased in the glioma cell derived-neurospheres. Similarly, also, glioma primary cell-derived neurospheres showed higher levels of NOS2 expression and activity when compared to the adherent tumor cells cultured in standard conditions [28]. SOX-2, which is a member of the SRY-related HMG-box (SOX) transcription factor family involved in several cellular processes, has been implicated in cancer development [48,49,50]. SOX-2 is considered a key factor in both tumorigenicity and drug resistance in GSCs. According to some authors, SOX-2 knockdown indeed strongly affects the sphere generation capacity in glioma cell cultures, the dedifferentiation rate, and the stemness phenotype, thus reducing tumorigenicity [25,51,52]. Of note, we recently and firstly showed a high and significant correlation between NOS2 and SOX-2 expression in glioma cells [28].Collectively, all of these studies, among others, suggest that NO signaling plays an important role in GSC maintenance and resistance to anticancer therapies. Thus, the NOS2/NO system as a biomarker for glioma and/or as a potential pharmacological target is extremely promising. In this context, the inhibition of NO production may have a significant therapeutic potential also to improve conventional treatment, even if the clinical studies are still limited [53]. A phase I open-label study was conducted to assess the combination of ASP9853, an inhibitor of NOS2 dimerization, which results in a decrease of NO production, plus docetaxel in patients with advanced solid tumors [54]. Based on the observed toxicities, the study was discontinued due to neutropenia. On the other hand, simple arginine derivatives, known as pan-NOS inhibitors (i.e., L-NMMA), were proven to be non toxic in different clinical trials as hemodynamic modulators [55]. Of note, a clinical phase 1b trial of L-NMMA plus docetaxel in the treatment of refractory locally advanced or metastatic triple negative breast cancer patients is in progress, and no dose-limiting toxicities have been reported to date [56].

In an effort to highlight the functional role of the NOS2/NO system in glioma biology, the purpose of the current study was to explore the effect of the NOS2 inhibitor 1400W at no cytotoxic concentrations toward the proliferation and migration rate, clonogenic potential, and neurosphere generation ability of glioma cells expressing high levels of NOS2. NOS2, which is basically expressed in the U-87 MG, T98G glioma cell lines and human glioma primary cells, was confirmed to be upmodulated in the relative neurospheres generated in specific culture conditions. Of note, 1400W, at an effective concentration to inhibit NO generation, was able to significantly reduce the proliferation, migration, colony formation, and neurosphere generation abilities of glioma cells, thus supporting the emerging relevance of NOS2 as a functional player in glioma biology. Results from experiments that aimed to investigate the effect of the NO chemical donor SNAP also support a functional role for NO in the glioma cell survival and proliferation rates as well as clonogenic potential. In addition, the methodological approaches used in this work to study proliferation and migration, together with those aimed at verifying the tumor potential of developing spheres, appear useful for the screening of new and increasingly specific NOS2 inhibitors such as acetamidines, which have been recently discovered and are structurally related to the 1400W [31]. In this context, our findings could represent a useful contribution to the development of potential therapeutic approaches for the treatment of glioma based on knowledge of the signaling pathways involved in the NO-mediated glioma cell regulation. At the same time, further studies are required to gain insights into the signaling networks involved in NOS2/NO system expression and overexpression, which in turn might underlie the abnormal expansion of GICs. 

## 4. Materials and Methods

### 4.1. GBM Cell Lines and Neurosphere Generation

U-87 MG and T98G, which are both human grade IV glioma cell lines, were purchased from the European Collection of Authenticated Cell Cultures (ECACC) and American Type Culture Collection (ATCC), respectively. Allen et al. reported the origin of the U-87 MG cell line as mislabeled and unknown; they compared the original U-87MG cell line firstly identified at Uppsala University with the commercial U-87MG cell line from ATCC HTB 14TM. Genotyping analysis revealed that the ATCC and Uppsala U-87 MG cell lines were from distinct origins [57]; however, other recent studies [58,59,60] have demonstrated that the U-87MG ATCC cell line has several typical characteristics of glioblastoma through analyses of cell morphology and gene expression profile, even though the origin patient is unknown. Thus, U-87 MG can be used as a *bonafide* human glioblastoma cell line. Here, we used a U-87 MG cell line (89081402) obtained from the European Collection of Authenticated Cell Cultures (ECACC) showing a DNA profile similar to U87 (ATCC). Adherent cell lines were cultured in DMEM (Dulbecco’s Modified Eagle Medium) supplemented with 10% (*v*/*v*) of fetal calf serum (FCS), 2 mM L-glutamine, 100 U/mL penicillin, and 100 mg/mL streptomycin (standard medium, St-M) within cell culture flasks (25 cm^2^) at a density of 15 × 10^3^/cm^2^ and incubated in sterile conditions at 37 °C in a 5% CO_2_ humidified atmosphere. After reaching 80% confluence, adherent cell cultures were expanded after previous detachment with trypsin solution from bovine pancreas. The complete medium was totally replaced every three days. At approximately 70% confluence, the cells were treated or not with *N*-(3-(aminomethyl)benzyl)acetamidine (1400W) (Sigma Chemical Co., Milan, Italy) (100 µM in PBS solution) for 24 h. PBS at the same volume was added to the control conditions. Afterward, cell number and viability were evaluated by trypan blue dye exclusion assay (0.04% in PBS) in a Bürker chamber by using an optical microscopy (Eclipse 50i, Nikon Corporation, Tokyo, Japan). The cell number of live and dead cells was registered. Where specified, cells were treated with an NO-donor S-nitroso-*N*-acetylpenicillamine (SNAP) (Cayman Chemical-Ann Arbor, MI, USA), as an exogenous NO source. SNAP was used at the concentration of 100 µM, as previously suggested [61]. Cell viability was also assessed by flow cytometric analysis, by incubating cells with propidium iodide (PI) for 30 min; then, both viable and dead cells were analyzed using a flow cytometer (BD Instruments Inc., San José, CA, USA), and the BD CellQuest Software program (BD Instruments Inc.)

To generate neurospheres, glioma cell lineswerekept in serum-free DMEM/F12 (1:1, *v/v*), a specific medium for brain tumor stem cell growth, containing 20 ng/mL of both recombinant human epidermal growth factor (EGF) and basic fibroblast growth factor (b-FGF), B27 supplement, penicillin/streptomycin, and glutamine (Glioma Stem Cell Medium, GSC-M), as previously described [28]. Cell cultures were incubated within 25 cm^2^ cell culture flasks at 37 °C in a humidified atmosphere with 5% CO_2_, in sterile conditions. The obtained neurosphere cultures were routinely evaluated by flow cytometry for stemness marker expression, as yet described [8]. In all of the experiments, used neurospheres showed a high positivity for β-tubulin (>60%), nestin (>80%), and SOX-2 (>60%). Neurospheres were daily treated or not with the NOS2 inhibitor 1400W at 100 µM, as previously suggested [24]. The morphology of cell lines cultured in St-M and derived neurospheres obtained in GSC-M was visualized and imaged by Nikon Eclipse TS100 at several time intervals of the experiment. Where not otherwise specified, the reagents and consumables were purchased from EuroClone (EuroClone, West York, UK). Neurosphere size was evaluated at 20 culture days in the absence or presence of 1400W (100 μM). Briefly, 10 bright field images, at 4× magnification, were randomly taken from each condition with an inverted microscope under phase contrast mode and analyzed using Image J software. The neurosphere average area, as expressed in mm^2^, was calculated dividing the entire neurosphere area by the total number of neurospheres. Each experiment was performed in duplicate. 

### 4.2. Glioma Primary Cell Culture and Neurosphere Generation

Glioma primary culture was obtained from a human solid biopsy with the diagnosis of Grade IV glioma (glioblastoma multiforme, GBM) of a patient affected by malignant glioma, as confirmed by neuropathological examination, who underwent a surgical exeresis, in accordance with fluorescence-guided tumor resection protocol (ALAPDD assisted resection). The enrolled GBM patient presented with a preoperative KPS (Karnofsky Performance Status) of 80. The tumor biopsy was positive for GFAP (glial fibrillary acidic protein), positive for the proliferative marker Ki67 (50%), and negative for IDH1 (isocitrate dehydrogenase) mutation. The study was approved by the Ethics Committee of the University of L’Aquila (n. 40070), and the patient gave written informed consent. Tumor biopsy was obtained from the Neurosurgery Unit, S. Salvatore Hospital, L’Aquila, Italy. Method for primary culture isolation was previously described [8]. Briefly, fresh surgical specimens were washed in PBS and mechanically manipulated by scalpel to remove blood cells and visible necrotic portions. To obtain single cell suspensions, mechanical and enzymatic tissue dissociation was performed by scalpel and trypsin solution at 37 °C for 15–20 min in a water bath by gentle stirring. Thus, the primary culture was expanded after reaching 80% confluence and was cultured in St-M to establish adherent cultures or GSC-M to generate neurospheres. The cells were incubated in sterile conditions at 37 °C in a 5% CO2 humidified atmosphere, and medium was totally replaced every three days. Cell number and viability were determined using the trypan blue dye, as above described for the glioma cell line, and the morphology was analyzed by Nikon Eclipse TS100 microscope (Nikon, Tokyo, Japan). The morphology, size, and phenotype of primary GBM culture derived-neurospheres was evaluated as above described for U-87 MG cells.

### 4.3. Western Blot

Cell pellets were collected and homogenized in ice-cold RIPA buffer (phosphate buffer saline pH 7.4 supplemented with 0.5% sodium deoxycolate, 1% NP40, 0.1% SDS, 5 mM of EDTA (Ethylenediaminetetraacetic acid), 100 mM of sodium fluoride, 2 mM of sodium pyrophosphate, 1 mM of PMSF (Phenylmethylsulfonyl fluoride), 2 mM of ortovanadate, 10 μg/mL of leupeptin, 10 μg/mL of aprotinin, 10 μg/mL of pepstatin). Homogenates were centrifuged at 600× *g* for 30 min at 4 °C, and the protein content was quantified into supernatants using the BCA protein assay kit (Pierce, Rockford, IL, USA). Samples (40 μg/lane) were run on 8.5% SDS polyacrylamide gels according to standard procedures, and proteins were transferred onto nitrocellulose membranes. Non-specific binding sites were blocked with 5% non-fat dry milk for 1 h at room temperature, and membranes were incubated overnight at 4 °C with primary antibody anti-NOS2 (Thermo Fisher Scientific, Waltham, Massachusetts, United States), and anti-β-actin (Bio-Rad, Hercules, CA, USA). Secondary antibodies, anti-rabbit IgG for NOS2 detection and anti-rabbit IgG for β-actin, were used, and immunoreactive bands were visualized by ECL chemiluminescent substrate reagent according to the manufacturer instructions and acquired by UVItec Alliance (Cambridge, UK). Densitometric analysis was performed by software provided by the company. Relative band intensity was normalized to respective β-actin bands. The murine macrophage cell line RAW 264.7 untreated or treated with LPS (1 µg/mL) and IFN-γ (100 ng/mL) for 24 h was used as the NOS2 negative and positive control, respectively.

### 4.4. Nitrite Level Assay

The enzymatic activity of NOS2 was evaluated by measuring nitrite levels using nitrate reductase and Griess reaction through a colorimetric assay (Nitrite Assay kit-Sigma-Aldrich Co., Milan, Italy). Glioma cells and relative-derived neurospheres were seeded as above described for different incubation times, after which nitrite levels were assayed in the cell supernatants applied to a 96-well microtiter plate, according to the manufacturer’s instructions. The absorbance was measured by spectrophotometric reading at 550 nm using a microplate reader (Bio-Rad, Hercules, CA, USA). The nitrite content of each sample was evaluated with a standard curve obtained by linear regression made with sodium nitrite and expressed in μg/mL. Each sample was assayed in duplicate.

### 4.5. Clonogenic Assay

The ability of the glioma cells to generate *in vitro* colonies was determined using clonogenic assay. Briefly, U-87 MG, T98G, and glioma primary cells were incubated in St-M in six-well plates at a concentration of 1.000 cells/well until colony formation. The medium was regularly changed. The cells were daily treated with 1400W (100 µM) or SNAP (100 µM). After 10 days for U-87 MG and T98G, and 15 days for glioma primary cells, the supernatants were removed; then, colonies were gently washed with PBS, fixed with cold methanol for 20 min, and stained with crystal violet 0.1% in PBS at room temperature for 10 min and air-dried. Images were captured and the total number of colonies/well was counted.

### 4.6. Cell Migration Assays

The effect of 1400W on glioma cell proliferation and migration was assessed using wound-healing assay, as previously described [62]. U-87 MG and T98G cells were plated at 6 × 10^4^/cm^2^ in six-well plates and cultured until reaching confluence. DMEM was removed and cell monolayers were scratched using a 200-μL pipet tip. Then, the cells were washed with PBS in order to remove debris, and cultures were incubated with fresh medium at 37 °C in a 5% CO2 humidified atmosphere in the absence or presence of 1400W (100 µM) or the NO chemical donor SNAP (100 µM). Images of cell migration were captured by an inverted light microscope (Eclipse TS 100, Nikon) (10× magnification) at different time points after the injury (0–36 h). The experiments were conducted in duplicate, and nine fields for each condition were analyzed. To calculate the percentage of wound closure, the images were analyzed quantitatively using the standalone TScratch software that automatically calculates the portion of area occupied by the cells by a mathematical model [63]. The quantification of a relative scratched monolayer closure (wound closure) was performed according to the equation where Tn is a specific time point after the scratching:

% Relative wound closure = [% of scratched area at T0–% of scratched area at Tn] (×100) [% of scratched area at T0]

### 4.7. Statistical Analysis

All of the data were analyzed using Prism 6.0 GraphPad Software (GraphPad, San Diego, CA, USA). Results are expressed as mean ± SEM from two or three independent experiments conducted in duplicate or triplicate, as specified. The results of the T98G cell line are presented as the mean ± SD of one experiment performed in duplicate. For comparison between two means, Student’s unpaired *t*-test was used. For comparisons of the mean values among groups, a one-way or repeated measures two-way ANOVA followed by Bonferroni post hoc test were used. Values of *p* less than 0.05 were accepted as significant.

## Abbreviations

GBM	Glioblastoma multiforme
GSCs	Glioma Stem Cells
GICs	Glioma Initiating Cells
NOS2	Nitric Oxide Synthase 2
NO	Nitric Oxide
1400W	*N*-(3-(Aminomethyl)benzyl)acetamidine
SNAP	S-nitroso-N-acetylpenicillamine
St-M	Standard Medium
GSC-M	Glioma Stem Cell Medium
KPS	Karnofsky Performance Status
GFAP	Glial fibrillary acidic protein
IDH1	Isocitrate dehydrogenase 1
TMZ	Temozolomide
l-NMMA	N^G^-Monomethyl-l-arginine
